# Eribulin, trastuzumab, and pertuzumab as first-line therapy for patients with HER2-positive metastatic breast cancer: a phase II, multicenter, collaborative, open-label, single-arm clinical trial

**DOI:** 10.1007/s10637-019-00755-x

**Published:** 2019-03-08

**Authors:** Kenichi Inoue, Jun Ninomiya, Tsuyoshi Saito, Katsuhiko Okubo, Takashi Nakakuma, Hirofumi Yamada, Kei Kimizuka, Tohru Higuchi, for the SBCCSG-36 investigators

**Affiliations:** 1 Division of Breast Oncology, Saitama Cancer Center, 780 Komuro, Ina-machi, Kita-adachi-gun, Saitama, 362-0806 Japan; 2 Department of Breast Surgery, Ninomiya Hospital, Soka, Japan; 3 Department of Breast Surgery, Saitama Red Cross Hospital, Saitama, Japan; 4 Department of Breast Unit, Toda Central General Hospital, Saitama, Japan; 5 Department of Breast Surgery, Ageo Central General Hospital, Ageo, Japan; 6 Department of Surgery, Sekishindo Hospital, Saitama, Japan; 7 Department of Breast Surgery, Kasukabe Medical Center, Kasukabe, Japan

**Keywords:** Metastatic breast cancer, Eribulin, Trastuzumab, Pertuzumab, HER2-positive, First-line therapy

## Abstract

*Purpose* To examine the efficacy and safety of triple therapy with eribulin, trastuzumab, and pertuzumab in patients with HER2-positive metastatic breast cancer (MBC) who never received any prior therapy in the first-line metastatic/advanced setting. *Methods* Eribulin 1.4 mg/m^2^ (days 1 and 8), trastuzumab 8 mg/kg over 90 min and 6 mg/kg over 30 min, and pertuzumab 840 mg/body over 60 min and 420 mg/body over 30 min were administered intravenously in 21-day cycles. *Results* 25 women (median age, 57 years [range, 41–75 years]) received a median of 10 cycles (range, 0–34 cycles); 24 had performance status (PS) 0, 1 PS 1, 8 stage IV breast cancer, and 17 recurrence. Lung and liver metastases occurred in 9 and 9 patients, respectively. Median time to treatment failure with eribulin was 9.1 months (95% confidence interval [CI], 4.3–13.9 months), and median progression-free survival was 23.1 months (95% CI, 14.4–31.8 months). The overall response rate (complete response [CR] + partial response [PR]) was 80.0% (95% CI, 59.3–93.2%), and the clinical benefit rate (CR + PR + stable disease ≥24 weeks) was 84.0% (95% CI, 63.9–95.5%). The most common treatment-emergent adverse events (TEAEs) were alopecia (92.0%), fatigue (68.0%), and sensory peripheral neuropathy (60.0%). Grade 3/4 TEAEs occurred in 11 patients (44.0%). The only grade 4 TEAE was neutrophil count decreased (16.0%). Neither grade 4 peripheral neuropathy nor febrile neutropenia occurred. *Conclusions* ETP therapy showed acceptable efficacy and safety and is a potential first-line therapy for patients with HER2-positive MBC.

## Introduction

The amplification of the human epidermal growth factor receptor 2 (HER2)/neu oncogene occurs in 25 to 30% of breast cancers, which increases the aggressiveness of the malignancy [1, 2]. Up to 5% of patients present with distal metastases at the time of diagnosis [3], and an additional 10 to 15% of patients will develop metastasis within 3 years after diagnosis [4]. Despite remarkable progress in the treatment of HER2-positive breast cancer, metastatic breast cancer (MBC) is still incurable in the majority of patients [5].

Two randomized clinical studies of trastuzumab in combination with conventional chemotherapy (doxorubicin, epirubicin, cyclophosphamide, or paclitaxel) [6] and a taxane (docetaxel) [7] showed that the combined therapy arms increased the response rates and extended the median time to treatment failure (TTF) and the median overall survival (OS) as compared with the control treatment arms. Therefore, the therapeutic regimen combining trastuzumab and a taxane was recommended for patients with HER2-positive MBC. Results from a phase III, placebo-controlled clinical trial of docetaxel-trastuzumab-pertuzumab (DTP) for patients with HER2-positive MBC (CLEOPATRA Study) [8] led to the approval of the triple regimen by the Food and Drug Administration (FDA) as the first “first-line” therapy. Additional confirmatory clinical data from the CLEOPATRA Study [9, 10] demonstrated the statistically significant and clinically meaningful survival benefit of DTP therapy; progression-free survival (PFS) assessed by the investigator and safety were consistent between the updated and primary analyses. Hence, DTP therapy is currently a well-established first-line therapy for this patient population. Nevertheless, docetaxel is often difficult to administer continuously due to its acute and/or cumulative toxicities (e.g., infusion reaction, febrile neutropenia, nail toxicities, fatigue, edema, rash, and peripheral neuropathy) [11].

Eribulin is a structurally simplified synthetic macrocyclic ketone analogue of halichondrin B [12] with a unique mechanism of action—binding to the high affinity sites on the growing plus (+) ends of microtubules appearing different from the taxane- and vinca-binding sites [13]—and showed potent anticancer activity and an acceptable safety profile in 7 phase II clinical trials for patients with MBC [14–19]. Furthermore, a global, multicenter, randomized, open-label, phase III clinical trial of eribulin (Study 305/EMBRACE) showed statistically significant and clinically meaningful improvements in OS compared to treatment of physician’s choice (most often including vinorelbine, gemcitabine, and capecitabine) in women with heavily pretreated MBC [20]. The landmark study led to the approval of eribulin by the FDA for the treatment of MBC in patients who had received at least two prior chemotherapy regimens. Another phase III clinical study of eribulin and capecitabine (Study 301) indicated the ORRs, PFSs, OSs, and overall quality of life (QOL) scores, all of which were comparable but without statistically significant differences between the study groups [21]. Furthermore, the pooled analysis of these two phase III studies confirmed the significant survival benefit of eribulin compared to control after treatment with a taxane and an anthracycline in patients with MBC [22]. Thus, eribulin showed therapeutic benefits in phase II/III clinical trials [14–22]. Furthermore, a retrospective observational study of eribulin in Japanese women with MBC [23]—which explored the extrapolation of these findings to the real-world clinical settings—suggested that the safety profile of eribulin in real-world clinical practice may be considered more acceptable than that reported in some of these studies.

Based on a wealth of clinical evidence mentioned above, we made a hypothesis that eribulin is superior to docetaxel in safety and tolerability and allows longer-term triple therapy. The objective of the present study was to examine the efficacy and safety of the eribulin-trastuzumab-pertuzumab (ETP) regimen as first-line therapy for patients with HER2-positive MBC.

## Methods

### Study design

This phase II, multicenter, collaborative, open-label, single-arm study was conducted to examine the usefulness of the ETP regimen as “first-line” therapy for patients with HER2-postive MBC who had inoperable breast cancer (stage IV) or recurrence after surgery at the time of initial visit. The study protocol was approved by the Institutional or Central Ethics Committee, and the study was conducted in accordance with the Declaration of Helsinki, Good Clinical Practice, as well as local ethical and legal regulations. All patients provided written informed consent before enrollment. The present study was registered (University Hospital Medical Information Network identifier: 000021585). The cutoff point for the data reported herein was 9 months following the enrollment of the last patient.

### Patients

Female patients were considered eligible for inclusion in the present study when meeting the following criteria: diagnosed with histologically invasive breast cancer; being ≥18 years old and having an expectable prognosis; HER2 overexpression (immunohistochemistry 3-positive or fluorescence in situ hybridization-positive) in the primary or metastatic tumor; first-line therapy for a patient who had inoperable breast cancer (stage IV) at the time of initial visit or recurrence after surgery; Eastern Cooperative Oncology Group [ECOG] performance status 0 or 1; a definite metastatic lesion that is assessed according to the Japanese version of the Response Evaluation Criteria in Solid Tumors (RECIST) version 1.1 [24]; conserved function of major organs; hematology and blood chemistry variables within 14 days prior to enrollment that meet all of the following levels— ≥ 1500/mm^3^ in neutrophil count, ≥75,000/mm^3^ in platelet count, ≥9.0 g/dL in hemoglobin, ≤2.0 mg/dL in total bilirubin, and <100 IU/L in aspartate aminotransferase and alanine aminotransferase, and <1.5 mg/dL in serum creatinine; no clinical problem in electrocardiography; ≥55% in left ventricular ejection fraction in electrocardiography; and written informed consent provided by the patient herself. Patients were excluded when falling under any of the following key criteria: being complicated by an infection or being suspected of an infection because of fever; severe drug allergy; severe renal or hepatic disorder; voluminous pleural effusion or ascites; being or suspected of being pregnant; active double cancer; metachronous or simultaneous bilateral breast cancer; active brain metastasis; and being assessed ineligible for the study by the investigator.

### Treatment

**Fig. 1:**
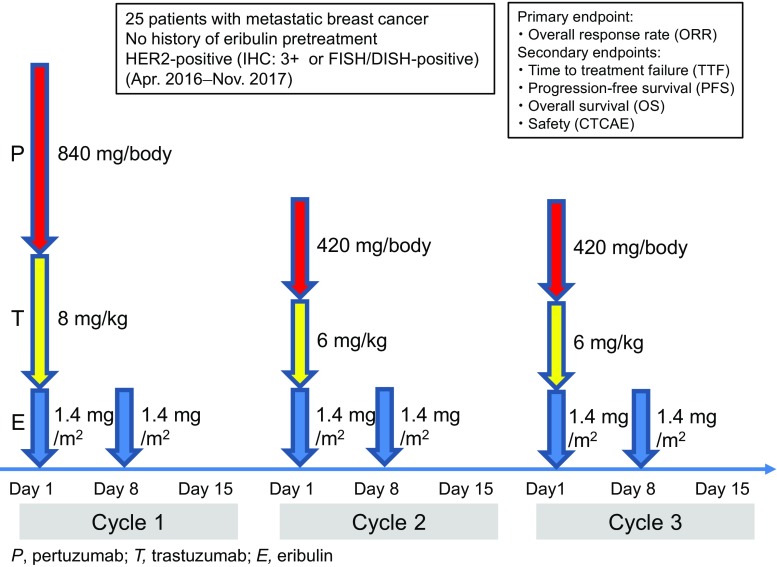
Dosing schedule of study drugs. HER2, human epidermal growth factor receptor 2; IHC, immunohistochemistry; FISH, fluorescence in situ hybridization; DISH, dual-color in situ hybridization; P, pertuzumab; T, trastuzumab; E, eribulin

Patients underwent study treatments as scheduled (Fig. 1). Concretely, patients received eribulin mesylate 1.4 mg/m^2^ as a 2- to 5-min intravenous (IV) infusion on days 1 and 8 of each 21-day cycle, trastuzumab 8 mg/kg as the initial 90-min IV infusion and 6 mg/kg as the second and subsequent 30-min IV infusions in each 21-day cycle, and pertuzumab 840 mg/body as the initial 60-min IV infusion and 420 mg/body as the second and subsequent 30-min IV infusions in each 21-day cycle. Treatment cycles consisting of 2 doses of eribulin and 7-day drug holiday were repeated until treatment discontinuation. Patients were treated with trastuzumab and pertuzumab when dose reductions of eribulin failed to resolve treatment-emergent adverse events (TEAEs). Two dose reductions (1.1 and 0.7 mg/m^2^) were permitted for eribulin, but not for trastuzumab and pertuzumab. Furthermore, patients were allowed to remain on study treatment until failure in obtaining additional clinical benefit, disease progression, occurrence of unacceptable AEs, or withdrawal of consent to study participation.

### Assessment of efficacy

The investigator assessed tumor lesions according to the Japanese version of the RECIST version 1.1 [24] at screening, at week 6 (±3 weeks) after the day of first dosing, and every 6 weeks (±3 weeks) from the prior date of tumor lesion assessment. The primary endpoint was the overall response rate (ORR: the proportion of patients who gained complete response [CR] plus those who gained partial response [PR]). CR was defined as the disappearance of all target lesions on the basis of results from imaging modalities (i.e., computed tomography and magnetic resonance imaging). PR was defined as a ≥ 30% reduction in the diameter sum of target lesions as compared with that found at baseline. Progressive disease (PD) was defined as a ≥ 20% increase in the diameter sum of target lesions as compared with the minimal diameter sum during the clinical course, and a ≥ 5 mm increase in the absolute value of diameter sum. The secondary endpoints were the time to treatment failure (TTF: a composite of disease progression, death, discontinuation of treatment, or switch to other types of antitumor therapy), progression-free survival (PFS: the time from enrollment to the first documented evidence of PD or death from any cause), and TEAEs of ETP therapy. In addition, the clinical benefit rate (CBR: CR + PR + long-term stable disease [LSD] ≥24 weeks) and the disease control rate (DCR: CR + PR + stable disease [SD]) were calculated. Best overall responses were determined and recorded, and percentage changes in the total sum of target lesion diameters were calculated.

### Safety

The investigator monitored and graded TEAEs according to the Japanese version of the National Cancer Institute Common Terminology Criteria for Adverse Events (CTCAE) version 4.03 [25] with respect to the prespecified safety valuables (e.g., periodic measurements of subjective symptoms, objective signs, physical examinations, vital signs, hematology, blood chemistry, and imaging modalities) at baseline, every 9 weeks after the onset of treatment, and at the time when study treatment was completed or discontinued. Furthermore, an independent efficacy and safety evaluation committee assessed the incidence and severity of TEAEs.

### Statistical analyses

Due to the small sample size, formal statistical analyzes were not planned for this study. In consideration of the results from the CLEOPATRA study [8–10], however, we expected an ORR of approximately 80% for ETP therapy. The target number of patients was calculated to be 16 based on the following statistical conditions: 90% confidence interval [CI]: 0.16, two-sided; duration of enrollment: 12 months; duration of follow-up to assess the OS after the completion of enrollment: 48 months: α level: 0.05; and power: 80%. SPSS version 19 (IBM, Armonk, NY) was used to make all statistical analyses.

## Results

### Patients

A total of 25 female patients (median age: 57 years [41–75]) were enrolled from April 18, 2016, through November 22, 2017, at 7 medical institutions in Japan. Demographic and clinical characteristics of patients at baseline are shown in Table 1. Among them, 24 had ECOG performance status (PS) 0, 1 PS 1, 8 stage IV breast cancer, and 17 recurrence after surgery. Anthracyclines, taxanes, and trastuzumab were administered in the neoadjuvant and adjuvant pharmacotherapies settings to 13, 15, and 14 patients, respectively. Overall, 48.0% (*n* = 12), 20.0% (*n* = 5), and 44.0% (*n* = 11) had estrogen receptor-, progesterone receptor-, and HER2-positive disease. The most common metastatic sites were the lung (36%, *n* = 9), liver (36%, *n* = 9), and bone (24%, *n* = 6).

**Table 1:** Demographic and clinical characteristics of patients at baseline (*N* = 25)

Characteristic	n	%
Age, years		
Median	57	
Range	41–75	
Status of menopause		
Premenopause	5	20.0
Postmenopause	20	80.0
Stage		
IV	8	32.0
Recurrence after surgery	17	68.0
ECOG performance status		
0	24	96.0
1	1	4.0
Histopathology		
Common type	25	100.0
ER status		
Positive	12	48.0
Negative	13	52.0
PgR status		
Positive	5	20.0
Negative	20	80.0
HER2 status		
Herceptest®/FISH or DISH		
3+/not evaluable	11	44.0
3+/positive	8	32.0
2+/positive	5	20.0
Not evaluable/positive	1	4.0
Prior adjuvant or neoadjuvant chemotherapy with anthracyclines		
Present	13	52.0
Absent	12	48.0
Prior adjuvant or neoadjuvant chemotherapy with taxanes		
Present	15	60.0
Absent	10	40.0
Prior adjuvant or neoadjuvant chemotherapy with trastuzumab		
Absent	11	44.0
Prior adjuvant or neoadjuvant hormone therapy		
Present	9	36.0
Absent	16	64.0
Current hormone therapy		
Present	2	8.0
Absent	23	92.0
Combination treatment with bone mineral modifiers		
Denosumab	3	12.0
Zolodronate	2	8.0
Metastatic sites		
3	8	32.0
4	2	8.0
Visceral metastases		
Present	14	56.0
Absent	11	44.0
Lung metastasis		
Present	9	36.0
Absent	16	64.0
Liver metastasis		
Present	9	36.0
Absent	16	64.0
Local lesion		
Present	12	48.0
Absent	13	52.0
Lymph node metastasis		
Present	16	64.0
Absent	9	36.0
Skin metastasis		
Present	3	12.0
Absent	22	88.0
Bone metastasis		
Present	6	24.0
Absent	19	76.0

*ECOG* Eastern Cooperative Oncology Group, *ER* estrogen receptor, *PgR* progesterone receptor, *HER2* human epidermal growth factor receptor 2, *FISH* fluorescence in situ hybridization, *DISH* dual-color in situ hybridization

### Study drug exposure

Patients received a median number of 10 cycles (range, 0–34) of treatment with eribulin during a median of 6.9 months of treatment (range, 2.1–26.2; Table 2). A reduction in eribulin dose from 1.4 to 1.1 mg/m^2^ after cycle 1 was required in 5 (20.0%) patients; among them, 3 (12.0%) patients required a further reduction to 0.7 mg/m^2^. Furthermore, 1 patient required a reduction in eribulin dose directly from 1.4 to 0.7 mg/m^2^ for the sake of safety. Dose omissions, reductions, delays, and interruptions occurred in 18, 6, 4, and 7 patients, respectively.

**Table 2:** Study drug exposure

	DTP (*N* = 25)
Number of cycles delivered	
Median (range) for trastuzumab	14.5 cycles (3–36)
Median (range) for pertuzumab	14.5 cycles (0–34)
Median (range) for eribulin	10.0 cycles (3–34)
Relative dose intensity (%)	
Median (range) for trastuzumab	97.1% (29.0–104.5)
Median (range) for pertuzumab	95.1% (0–104.5)
Median (range) for eribulin	96.4% (23.3–115.4)
Dose omissions*, n (%)	18 (72.0%)
Dose reductions, n (%)	6 (24.0%)
Dose delays, n (%)	4 (16.0%)
Dose interruptions**, n (%)	7 (28.0%)

*The same patients had several reasons

**The same patients had several adverse events

### Efficacy

**Fig. 2:**
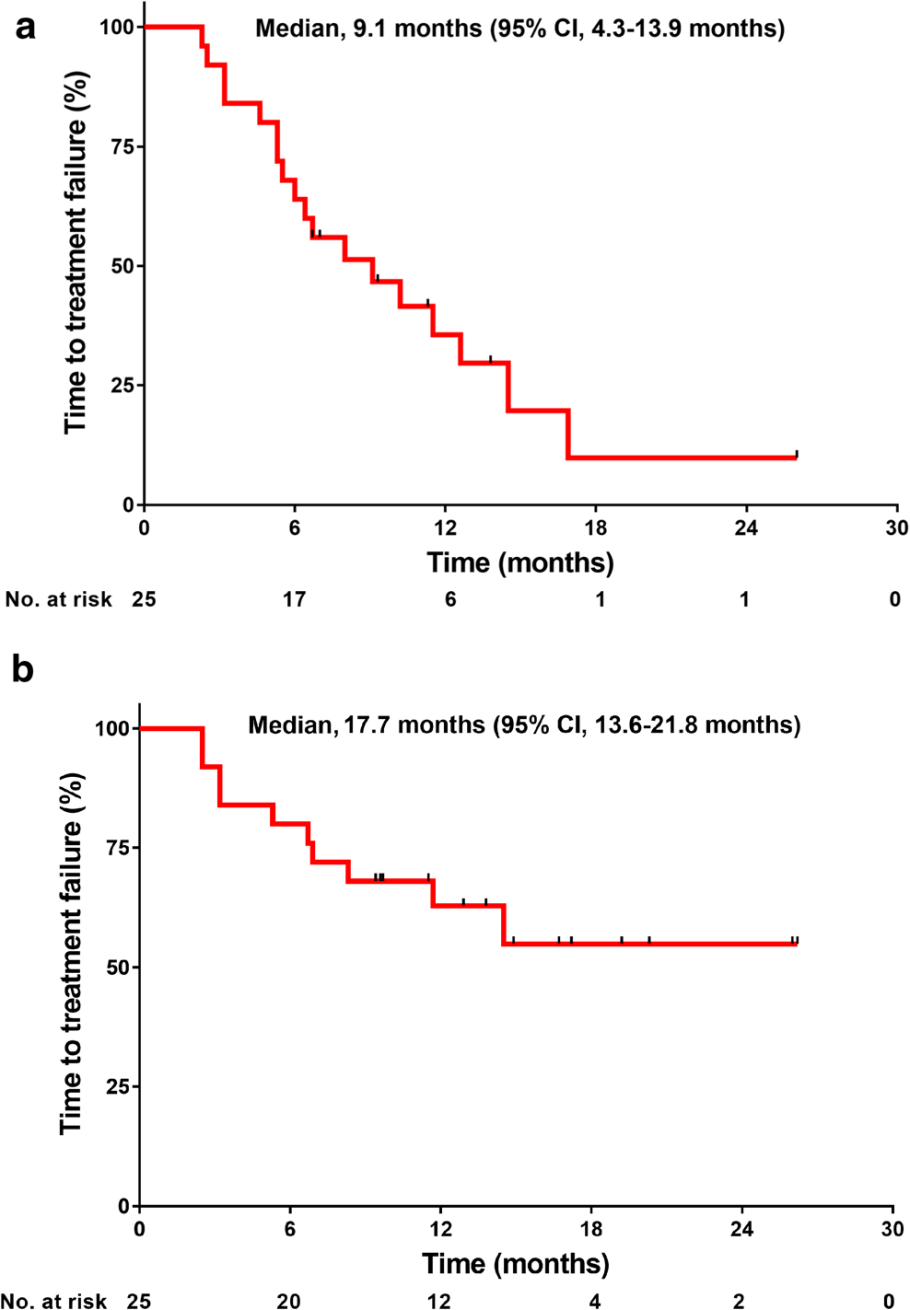
**a** Time to treatment failure with eribulin. **b** Time to treatment failure with trastuzumab and pertuzumab

**Fig. 3:**
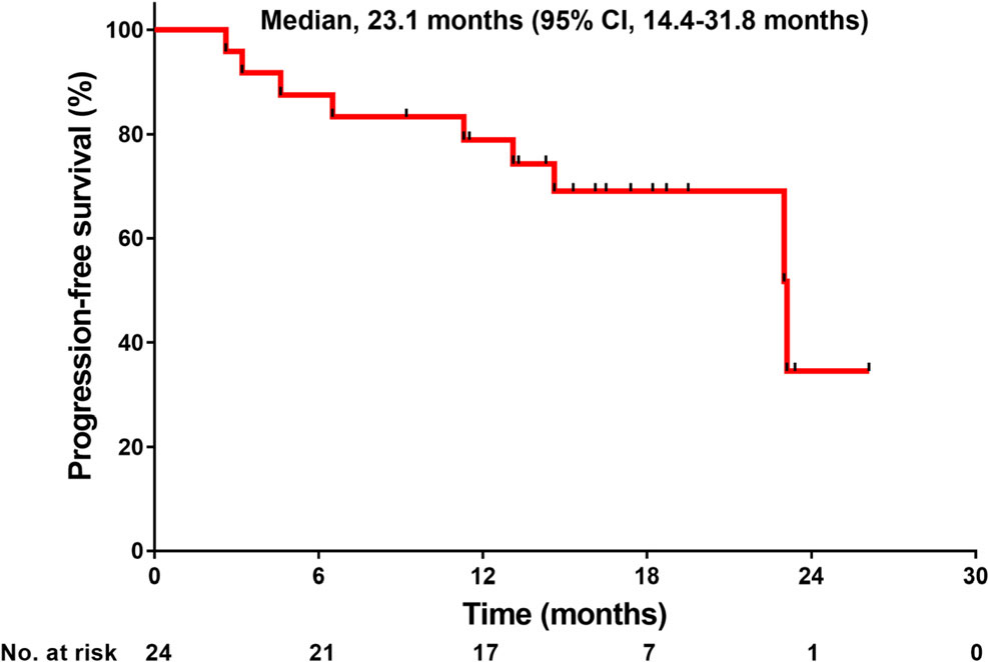
Progression-free survival

**Fig. 4:**
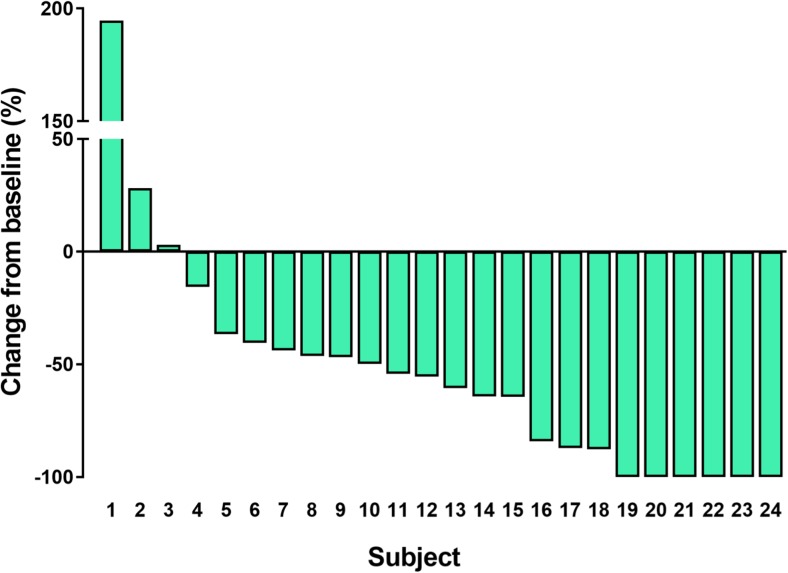
Waterfall plots of percentage changes in the total sum of the longest single diameter for measurable target lesions from baseline to the maximal tumor shrinkage regarding best overall responses: overall target lesions including the liver, lung, and others

With the exception of 1 patient who was not evaluable due to the grade 3 infusion reaction of pertuzumab, 24 were evaluable for best overall responses (Table 3). The ORR was 80.0% (95% CI, 59.3–93.2%). Furthermore, 3 (12.0%), 17 (68.0%), 1 (4.0%), 1 (4.0%), and 2 (8.0%) patients showed CR, PR, long-term stable disease (LSD), stable disease (SD), and PD, respectively. The median TTF with eribulin was 9.1 months (95% CI, 4.3–13.9 months; Fig. 2a), and the median TTF with trastuzumab and pertuzumab was 17.7 months (95% CI, 13.6–21.8 months (Fig. 2b). The median PFS was 23.1 months (95% CI, 14.4–31.8 months; Fig. 3). The CBR was 84.0% (95% CI, 63.9–95.5%). The disease control rate (DCR)—defined as the summed proportion of patients who achieved CR, PR, LSD, and SD—was 88.0% (95% CI: 68.8–97.5%). Twenty-one (87.5%) among 24 evaluable patients showed reductions in the diameters of overall target lesions including the liver, lung, and others (Fig. 4).

**Table 3:** Efficacy outcomes

Best overall response	All (*N* = 25)	Percent	95% CI
CR	3	12.0	2.6–31.2
PR	17	68.0	46.5–85.1
LSD	1	4.0	0.1–20.4
SD	1	4.0	0.1–20.4
PD	2	8.0	1.0–26.0
NE	1	4.0	0.1–20.4
ORR (CR + PR)	20	80.0	59.3–93.2
CBR (CR + PR + LSD)	21	84.0	63.9–95.5
DCR (CR + PR + LSD + SD)	22	88.0	68.8–97.5

*CI* confidence interval, *CR* complete response, *PR* partial response, *LSD* long-term stable disease ≥24 weeks, *SD* stable disease <24 weeks, *PD* progressive disease, *NE* not evaluable, *ORR* overall response rate, *DCR* disease control rate, *CBR* clinical benefit rate

### Safety

TEAEs occurred in all patients (Table 4). The most common TEAEs (all grades occurring in ≥40% of patients) were alopecia (92.0%, *n* = 23), fatigue (68.0%, *n* = 17), sensory peripheral neuropathy (60.0%, *n* = 15), anorexia (48.0%, *n* = 12), anemia, (44.0%, *n* = 11), and white blood cell count decreased (40.0%, *n* = 10). Grade 3/4 TEAEs occurred in 11 (44.0%) patients. The most common grade 3 TEAEs (occurring in ≥10.0% of patients) were white blood cell count decreased, neutrophil count decreased, and anemia (12.0% each, *n* = 3). The only grade 4 TEAE observed was neutrophil count decreased (16.0%, *n* = 4); none of these patients required treatment with granulocyte colony-stimulating factors. No febrile neutropenia or cardiac dysfunction occurred. Six patients experienced disease progression, 3 of whom died.

**Table 4:** Treatment-emergent adverse events by CTCAE grade in the safety population*

TEAEs	Severity, N (%)				
	All grades	Grade 1	Grade 2	Grade 3	Grade 4
Hematologic toxicities (*n* = 25)					
White blood cell count decreased	10(40.0)	3(12.0)	4(16.0)	3(12.0)	0(0)
Neutrophil count decreased	8(32.0)	1(4.0)	0(0)	3(12.0)	4(16.0)
Anemia	11(44.0)	7(28.0)	1(4.0)	3(12.0)	0(0)
Aspartate aminotransferase increased	6(24.0)	4(16.0)	2(8.0)	0(0)	0(0)
Alanine aminotransferase increased	7(28.0)	4(16.0)	3(120)	0(0)	0(0)
Blood bilirubin increased	3(12.0)	1(4.0)	2(8.0)	0(0)	0(0)
Creatinine increased	5(20.0)	4(16.0)	1(4.0)	0(0)	0(0)
Platelet count decreased	0(0)	0(0)	0(0)	0(0)	0(0)
Nonhematologic toxicities (*n* = 25)					
Alopecia	23(92.0)	13(52.0)	10(40.0)	–	–
Fatigue	17(68.0)	8(32.0)	7(28.0)	2(8.0)	0(0)
Sensory peripheral neuropathy	15(60.0)	12(48.0)	2(8.0)	1(4.0)	0(0)
Anorexia	12(48.0)	7(28.0)	4(16.0)	1(4.0)	0(0)
Mucositis oral	9(36.0)	9(36.0)	0(0)	0(0)	0(0)
Motor peripheral neuropathy	8(32.0)	4(16.0)	4(16.0)	0(0)	0(0)
Diarrhea	7(28.0)	5(20.0)	2(8.0)	0(0)	0(0)
Arthralgia	6(24.0)	4(16.0)	2(8.0)	0(0)	0(0)
Myalgia	5(20.0)	4(16.0)	0(0)	1(4.0)	0(0)
Nausea	5(20.0)	5(20.0)	0(0)	0(0)	0(0)
Nail discoloration	4(16.0)	4(16.0)	–	–	–
Dysgeusia	4(16.0)	2(8.0)	2(8.0)	–	–
Rash	3(12.0)	3(12.0)	0(0)	0(0)	0(0)
Vomiting	3(12.0)	3(12.0)	0(0)	0(0)	0(0)
Edema limbs	3(12.0)	3(12.0)	0(0)	0(0)	0(0)
Lung infection	1(4.0)	0(0)	0(0)	1(4.0)	0(0)
Infusion-related reaction by pertuzumab	1(4.0)	0(0)	0(0)	1(4.0)	0(0)
Constipation	1(4.0)	1(4.0)	0(0)	0(0)	0(0)
Epistaxis	1(4.0)	1(4.0)	0(0)	0(0)	0(0)
Olfactory nerve disorder	1(4.0)	1(4.0)	0(0)	0(0)	0(0)
Nail loss	1(4.0)	1(4.0)	0(0)	–	–

*The safety population consisted of patients who received at least one dose of the study drugs

*CTCAE* common terminology criteria for adverse events

## Discussion

This is the first phase II clinical trial of ETP therapy for patients with HER2-positive MBC and exhibited the efficacy variables that were equivalent to those of the pivotal study CLEOPATRA study [8]: an ORR of 80.0% (*n* = 20) vs. 80.2% (*n* = 275), the median PFS of 23.1 months vs. 18.5 months, and the number of cycles of 10 vs. 8. We speculate that these clinical outcomes are attributable to the following facts: 1) this study was designed as “first-line” therapy for this patient population; 2) eribulin, a drug with well-demonstrated efficacy in clinical studies, was administered to patients who were naïve to the drug; and 3) drug exposure as assessed by the number of cycles was greater to eribulin than to docetaxel. The incidence of neutrophil count decreased (all grades) was markedly lower in our study (32.0%) than in the CLEOPATRA study (52.8%). The median RDI for eribulin was 96.4%, indicating that most patients received the planned dose of 1.4 mg/m^2^ on days 1 and 8 of each 21-day cycle. ETP therapy caused grade 3/4 neutrophil count decreased (28.0%) without causing febrile neutropenia. In general, severe (grade 3/4) peripheral neuropathy occurs in up to 30% of patients who are treated with microtubule-targeting chemotherapy agents including taxanes and epothilones [26]. Therefore, the facts that the incidences of grade 3 sensory peripheral neuropathy, grade 4 sensory and motor peripheral neuropathies, as well as febrile neutropenia were as low as 4% (1/25 patients), 0% (0/25 patients), 0% (0/25 patients), respectively, are of particular note when considering the potential contribution of 10 cycles [median] of eribulin to cumulative neuropathic toxicities as compared with a median of 8 cycles of docetaxel in the CLEOPATRA study [8]. These findings are in line with the study of Vahdat et al. [14] that reported 9.8% and 20.0% in the incidences of grade 3 peripheral neuropathy for eribulin and ixabepilone—a microtubule stabilizer derived from epothilone B, respectively, and no case of grade 4 peripheral neuropathy for both drugs in the fewer median numbers of 5.0 and 3.5 cycles, respectively.

DTP therapy is recommended by the National Comprehensive Cancer Network guidelines as preferred first-line therapy for the treatment of patients with HER2-positive MBC [27]. The guidelines list the combinations of trastuzumab plus paclitaxel ± carboplatin, docetaxel, vinorelbine, and capecitabine as first-line treatment for HER2-positive disease. Docetaxel, one of the most active chemotherapeutic agents used in the treatment of MBC [28] and forms the taxane component of the standard treatment for patients with HER2-positive MBC, often impedes the triple therapy due to various reasons (e.g., a history of taxane allergy, resistance/refractoriness to or intolerance of taxanes, and acute/cumulative toxicities) [11]. Therefore, an effective and less toxic chemotherapy regimen combining a nontaxane drug with trastuzumab and pertuzumab is required as a therapeutic option for some patients with such inconveniences.

Eribulin, a novel nontaxane compound derived from halichondrin B, offers the following clinical advantages: 1) a rapid infusion time as short as 2 to 5 min; 2) no need for the use of solvents that taxanes necessitate (polysorbate 80 for docetaxel and Cremophor® EL for paclitaxel)—a procedure responsible for hypersensitivity reactions [13], thus leading to the avoidance of steroid premedication and to prevent the reactions; and 3) good efficacy and tolerability for heavily pretreated patients who have MBC and well-defined taxane resistance [29]. Based on the favorable tolerability of eribulin in phase I studies for various malignancies (e.g., lung, breast, colorectal cancers), the efficacy and safety of eribulin alone or in combination with various anticancer agents for patients with MBC have been investigated in a number of phase II [14–19] and III [20–22] studies. Results from these previous clinical studies have indicated the following efficacy variables: ORRs ranging from 9.3 to 80.2%; median PFSs ranging from 2.6 to 18.7 months; median OSs ranging from 9.0 to 56.5 months; and DCRs ranging 55.3 to 96.2%.

To date, furthermore, a diversity of first-line therapeutic regimens have been explored for patients with HER2-positive MBC. In a randomized phase 3 clinical study of trastuzumab monotherapy followed by trastuzumab plus docetaxel versus trastuzumab plus docetaxel [30], we demonstrated that the concurrent administration of trastuzumab and docetaxel was superior in OS to the sequential administration of trastuzumab followed by trastuzumab plus docetaxel. Several clinical studies have shown the efficacy and safety of trastuzumab and/or pertuzumab in combination with chemotherapeutic agents including docetaxel, paclitaxel, vinorelbine, and capecitabine in patients with HER2-positive MBC [6–8, 31–34]. Recently, Sakaguchi et al. conducted a multicenter, single-arm, phase II clinical trial of first-line eribulin and trastuzumab in 28 Japanese women with naïve progressive or recurrent HER2-positive breast cancer [35]. Patients received a median of 12 cycles (range, 2–53 cycles). They reported the following efficacy variables: the ORR, 53.6% (90% CI, 36.6–69.9); the CBR, 64.0% (90% CI, 45.61–79.76); and the median PFS, 344 days (95% CI, 237–680 days). In consideration of these data, namely, ours (ORR, 80.0%; CBR, 84.0%; and; and median PFS, 23.1 months) exhibited the improving-effect of pertuzumab on the efficacy variables. Grade 3/4 AEs occurred in 42.9% of patients. The most common AEs were sensory peripheral neuropathy (67.9%) and white blood cell count decreased (50.0%). They concluded that the eribulin-trastuzumab combination is a potentially important first-line option for advanced and recurrent HER2-positive breast cancer. In addition, Tono et al. conducted a single center feasibility study of ETP therapy in 10 Japanese women with previously treated advanced HER2-positive breast cancer to investigate the safety of ETP therapy and the QOL of patients, as well as to analyze biomarkers (e.g., serum HER2 extracellular domain [sHER] levels, PIK3CA gene mutation status, and circulating peripheral regulatory T cell levels) [36]. The median PFS was 4.8 months (95% CI, 3.7–5.9 months), the ORR was 20.0%, and the DCR was 70.0%. The most common grade 3 AEs were leukopenia and neutropenia (70.0%, respectively), and grade 4/5 AEs were not observed. The QOL scores exhibited an improvement trend at 3 months of the therapy. They found a strong association between the baseline sHER level and the serum trastuzumab trough concentration at 3 months of the therapy and concluded that ETP therapy might be a feasible option for patients with HER2-positive MBC that has wild-type or mutated PIK3CA. These most recent clinical studies, published in 2018, provide clinical evidence on the efficacy and safety of the double and triple chemotherapy regimens of eribulin in combination with trastuzumab ± pertuzumab, thus serving to strengthen the rationale and clinical relevance of ETP therapy for patients with HER2-positive MBC.

The present study has several limitations. First, sample size was small because of its nature of being a phase 2 clinical trial; however, the enrolled number of patients exceeded the scheduled number of patients—16 and was good enough for the analyses of efficacy variables. Second, the survival data were not mature at the time of clinical data cutoff. Nevertheless, we consider that the obtained data on the efficacy and safety of ETP therapy are sufficient as the preliminary clinical data to consider its potential of becoming an alternative regimen for the well-established first-line therapy using DTP.

## Conclusions

Triple therapy consisting of eribulin, trastuzumab, and pertuzumab provided a high ORR, a prolonged PFS, and an acceptable safety profile. Therefore, ETP therapy is a potentially important first-line therapy for patients with HER2-positive MBC. EMERALD, a phase III taxane-controlled clinical trial of eribulin, trastuzumab, and pertuzumab in Japan, is ongoing.
